# Seasonal variation in lifestyle behavior in Poland: Google searches and market sales analysis

**DOI:** 10.1186/s12889-021-11543-9

**Published:** 2021-08-06

**Authors:** Mikołaj Kamiński, Matylda Kręgielska-Narożna, Paweł Bogdański

**Affiliations:** Department of the Treatment of Obesity and Metabolic Disorders, and of Clinical Dietetics, Poznań University of Medical Sciences, Szamarzewskiego 82/84, 60-569 Poznań, Poland

**Keywords:** Consumption, Cigarettes, Alcohol, Diet, Google trends, Sales, Seasonality, Poland, Running, Lifestyle

## Abstract

**Background:**

Detection of the seasonal patterns of healthy and unhealthy behavior could be helpful for designing individual and population health interventions programs. This study investigates the seasonal variation in sales of common types of products in Poland and Polish Google queries related to healthy behavior.

**Methods:**

Data of index sales from a large Polish retail store franchise, from January 2014 to August 2019, has been analyzed. The commercial data included twelve types of products. The interest of Google users was investigated using Google Trends statistics for the same period for six lifestyle-related topics. The seasonality was checked using time series analysis.

**Results:**

Six of the consumer goods (dairy, ready-made meals, salty snacks, meats, beer, and cigarettes) were most commonly purchased in summer months, four (processed fish, food fats, wine, and alcohol 30%+) in December, and two (bread and sweets) in October. The lowest sales indexes were observed mostly in February. The interest in four topics that have been analyzed (“Diet,” “Dietitian,” “Weight loss,” and “Gym”), was highest in January, while interest in “Dietary supplements” was high in February, and “Running” in May. The search volume of the Google topics were the lowest in December.

**Conclusion:**

The purchase of food, drinks, and cigarettes, and the interest in information regarding different components of a healthy lifestyle has seasonal variation. New Year and Lent might be good periods to encourage healthy behavior. The motivation may decrease in summer and during Christmas.

**Supplementary Information:**

The online version contains supplementary material available at 10.1186/s12889-021-11543-9.

## Background

Lifestyle is the most important factor that has an impact on health and life expectancy [1, 2]. Lifestyle modifications can be considered as primary or secondary prevention. However, it has been observed that lifestyle recommendations are rarely followed [3, 4]. According to Eurostat, Poles consume fewer daily portions of fruit and vegetables [5] and spend less time on non-work-related physical activities [6] than EU-28 average. Furthermore, a large number of Polish adults are overweight or obese [7] and a higher proportion of citizens smoke cigarettes [8] as compared to the rest of the EU-28 countries. Therefore, there is an emerging need for efficient health promotion campaigns [9]. Rössner et al. established that New Year’s resolutions might be a strong motivator for weight loss [10]. This study hypothesizes that investigation of lifestyle behavior of the population may assist in identifying the periods when people are motivated to change. It is also important to identify the periods when unhealthy behavior is observed more frequently, to alert people at risk of the loss of motivation. Such knowledge may be useful for individuals practices and to design public health campaigns.

Longitudinal data of market sales and search engine queries could be useful in identifying the seasonal variation of lifestyle components. Purchase patterns have been used to determine the period of the highest consumption of cigarettes [11, 12] and alcohol [13]. Globally, Google is the most commonly used search engine [14], thus, most of the studies that have investigated web queries process data from this engine [15]. Patel et al. established that searches regarding diseases associated with lifestyle have seasonal and geographical variations [16, 17]. Kamiński et al. found that the interest of Google users in different types of diets is the lowest in December and surges in January and this may be attributed to New Year’s resolutions [18]. In the United States, searches associated with exercise are the highest during winter months, while those about weight loss are the highest in winter and summer season [19]. However, studies have primarily focused on limited types of searches representing lifestyle behaviors. The question is that do sales of consumer goods and interest in a healthy lifestyle have seasonal cycles.

This study investigates the seasonal variation in sales of common types of products in Poland and the Polish Google queries related to healthy behaviors to identify periods during the year that may be optimal for lifestyle interventions.

## Methods

This study utilizes market data and Google statistics. Therefore, the Ethical Committee’s approval was not required.

The market data was shared by a large Polish chain of retails stores. The name of the chain has been disclosed to the Editor-in-Chief. The entire chain represents 10 to 15% of the convenience store segment in Poland. In September 2019, dataset as monthly sales per shop, from January 2014 to August 2019, was collated. The data represents sales indexes from thousands of facilities. The number of active stores increased from 3204 in January 2014 to 5629 in August 2019. The sales statistics for bread, dairy products, sweets, processed fish, ready-made meals, salty snacks, food fats (edible fats e.g. butter, margarine, and oil), meats, beer, wine, alcohol with above 30% alcohol per volume, and cigarettes, were collated. The data points were the following months. Data was collated as an index where, if the value is equal to 100 it implies the mean sales of a group of products per shop in the first month in the dataset, that is January 2014.

In Poland, Google is the most popular search engine and is used by 93 to 98% of Internet users [20]. Google search statistics, for this study, have been collated from Google Trends (GT) (https://trends.google.com/trends/). GT defines the interest in a chosen phrase using an index called relative search volume (RSV). RSV is an index of Google queries that is adjusted to the number of search engine users in a given region. The value of RSV ranges from 0 to 100, and a value of 100 represents the peak of popularity (100% popularity in given period and location) and 0 indicates the absolute lack of popularity [15]. GT normalizes search results to the time and location of a query [21]. Location is established by IP address, GPS or by past activities [22]. GT identifies the “search terms,” and “topics.” Search terms show matches for all terms in a query in the language used, while topics are a group of concepts that may be proposed by GT for a given search term [23]. Topics are designed for comparison of given terms across all regions and all available languages. Therefore, matching topics in a chosen language is the same as having the option of all languages activated.

The researchers (MK and MKN) created an initial list of terms related to healthy lifestyle including, “gym” (pl. “siłownia”), “running” (pl. “bieganie”), “weight loss” (pl. “utrata wagi”), “dietitian” (pl. “dietetyk”), “dietary supplements” (pl. “suplementy diety”), “dietetic products” (pl. “produkty dietetyczne”), “healthy diet” (pl. “zdrowia dieta”), and “healthy lifestyle” (pl. “zdrowy styl życia”). This study matched the following topics: “Gym” (pl. “Siłownia”), “Running” (pl. “Bieganie”), “Weight loss” (pl. “Utrata wagi”), “Dietitian” (pl. “Dietetyk”), “Dietary supplements” (pl. “Suplementy diety”), and healthy diet (matched: “Diet” [pl. “Dieta”]). The data were collated for Poland, for the period from January 2014 to August 2019. The data points were the following months. All search conditions are presented in Supplementary Table 1, which are modified according to the Nuti et al. protocol for studies processing GT data [15]. In Poland, the seasons are as following: spring (March, April, May), summer (June, July, August), autumn (September, October, November), and winter (December, January, February).

Data analysis was conducted in March 2020. To detect significant seasonal variations, an exponential smoothing state-space model with Box-Cox transformation, autoregressive-moving average errors, trend, and seasonal components (TBATS) by using the *forecast* package version 8.9 of R [24], was fitted to the time trend. Seasonality is considered significant if the TBATS model has a seasonal component (otherwise the seasonal period is null). For e.g., if the data point represents months, the seasonal period is equal to 12 and implies that the applied model is best fitted when the yearly seasonal variation is considered. The seasonal component has been extracted using the Seasonal Decomposition of Time Series by Loess (Local Polynomial Regression Fitting). The yearly amplitude has been calculated as the difference between maximal and minimal seasonal components of the decomposed time series.

Additionally, we performed secular trend analysis. *p*-value < 0.05 in the Seasonal Mann-Kendall test from *Kendall* R package indicates a significant secular trend [23]. We performed a univariate linear regression to estimate slope expressed as changes of the index of sales per year or RSV per year for all significant secular trends.

## Results

All the analyzed groups of food, drinks, and cigarettes, and the topics, displayed seasonal variations (Table 1).

**Table 1:** Seasonal variations analysis for group of consumer products and Google Trends topic

Topic	TBATS (seasonality present, period [months])	Month with the highest seasonal component [Index/RSV]	Month with the lowest seasonal component [Index/RSV]	Seasonal component amplitude [Index/RSV]
** Group of food and drinks**				
Bread	YES, 12	October (8.80)	February (−11.53)	20.33
Dairy	YES, 12	August (8.57)	February (−13.57)	22.14
Sweets	YES, 12	October (13.10)	February (−11.31)	24.41
Processed fish	YES, 12	December (19.22)	April (−12.11)	31.32
Ready-made meals	YES, 12	June (9.69)	February (−13.04)	22.74
Salty snacks	YES, 12	June (17.44)	February (−17.49)	34.93
Food fats	YES, 12	December (13.93)	April (−18.89)	32.81
Meats	YES, 12	August (17.99)	February (−19.19)	37.18
Beer	YES, 12	August (24.23)	February (−24.00)	48.23
Wine	YES, 12	December (37.15)	February (−16.22)	53.37
Alcohol 30%+	YES, 12	December (14.02)	February (−7.52)	21.53
Cigarettes	YES, 12	August (10.80)	February (−15.36)	26.16
** Google Trends topics**				
Diet	YES, 12	February (14.47)	December (−18.22)	32.69
Dietetic	YES, 12	January (12.14)	December (−20.31)	32.45
Dietary supplements	YES, 12	February (13.98)	December (−9.64)	23.63
Weight loss	YES, 12	January (16.79)	December (−20.57)	37.37
Gym	YES, 12	January (21.99)	June (−14.45)	36.44
Running	YES, 12	May (32.17)	January (−19.23)	51.40

**Fig. 1:**
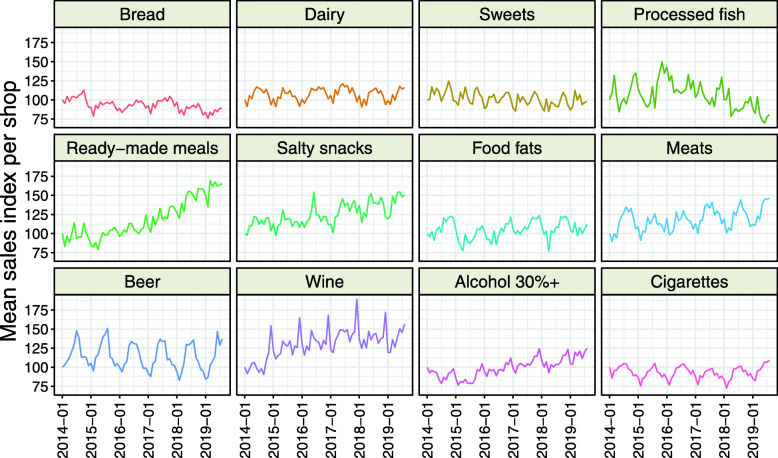
Time trends of mean sales index per shop of different groups of food and drinks

Six of the consumer goods (“dairy,” “ready-made meals,” “salty snacks,” “meats,” “beer,” and “cigarettes”) were most commonly purchased in summer months, four (“processed fish,” “food fats,” “wine,” and “alcohol 30%+”) in December, and two (“bread” and “sweets”) in October (Table 1, Fig. 1). The lowest sales indexes were observed mostly in February (*n* = 10). The highest early amplitude of interest was observed for “beer” and “wine.”

**Fig. 2:**
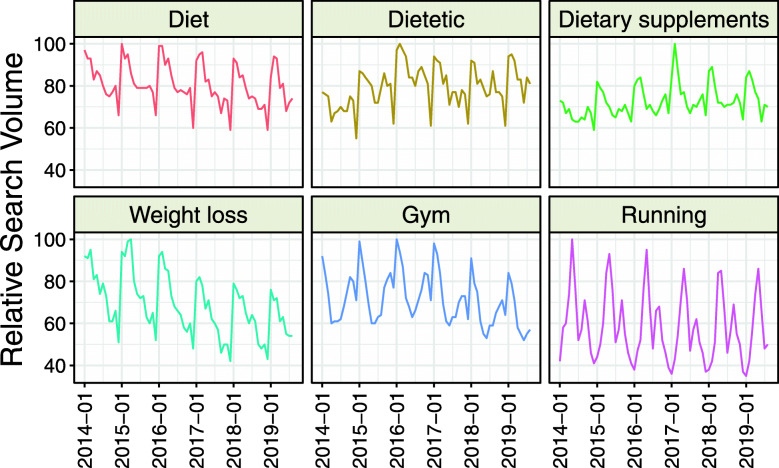
Time trends for Relative Search Volumes of Google Trends topics related to lifestyle

The interest in three (“Dietitian,” “Weight loss,” and “Gyms”) analyzed topics was the highest in January, two (“Diet” and “Dietary supplements”) in February, and one (“Running”) in May (Table 1, Fig. 2). The following topics: “Dietitian,” “Gym,” and “Running” revealed second peaks of interest in September, November, and August, respectively. The searches on lifestyle-related topics (“Diet,” “Dietitian,” “Dietary supplements,” “Weight loss”) were the lowest in December. The highest amplitude was observed for “Running.”

All goods and Google topics revealed a significant change in purchases or interest over time, while the purchase of “dairy,” “cigarettes,” and interest in “Running” did not changed in the analyzed period (Table 2). The most dynamic increase in sales was observed for “ready-made meals” (+ 13.74 sales index/year), “wine,” (+ 7.26 sales index/year), and “salty snacks” (+ 6.67 sales index/year), while the most dynamic decrease of sales was observed for “processed fish,” (− 5.00 sales index/year) and “bread” (− 2.45 sales index/year). Among analyzed Google topics, the interest in “Dietitian” (+ 1.45 RSV/year) and “Dietary supplements” (+ 1.40 RSV/year) increased over time, while the interest in other topics decreases in the analyzed period, particularly for “Weight loss” (− 4.78 RSV/year).

**Table 2:** Secular trends analysis for group of consumer products and Google Trends topic

Topic	Seasonal Mann-Kendall test	Slope [Index/year or RSV/year]
** Group of food and drinks**		
Bread	tau = −0.54; ***	−2.45; ***
Dairy	tau = 0.05; 0.64	–
Sweets	tau = − 0.59; ***	−2.20; ***
Processed fish	tau = − 0.49; ***	−5.00; ***
Ready-made meals	tau = 0.84; ***	13.74; ***
Salty snacks	tau = 0.81; ***	6.67; ***
Food fats	tau = 0.34; **	1.69; 0.045
Meats	tau = 0.61; ***	3.82; ***
Beer	tau = −0.39; ***	−1.02; 0.42
Wine	tau = 0.70; ***	7.26; ***
Alcohol 30%+	tau = 0.76; ***	5.83; ***
Cigarettes	tau = −0.07; 0.48	–
** Google Trends topics**		
Diet	tau = −0.58; ***	−1.93; **
Dietitian	tau = 0.33; **	1.45; 0.046
Dietary supplements	tau = 0.59; ***	1.40; 0.013
Weight loss	tau = −0.81; ***	−4.78; ***
Gym	tau = −0.48; ***	−2.42; **
Running	tau = − 0.58; ***	−1.34; 0.26

*RSV* Research Search Volume

** *p* < 0.01; *** *p* < 0.001

## Discussion

### Main findings

As the study is regarding seasonal variations, the major changes in the scope of the following months, from January to December, is discussed.

January is a period of both, New Year’s resolution and the carnival period. The first social phenomenon might enhance an individual’s motivation, while the second might attenuate it. A rapid increase in Google searches related to diet, dieticians, dietary supplements, weight loss, and gym, was observed at this time. Cherchye et al. observed that the consumption of healthy foods surges globally in January and gradually decreases over the year [25]. Reports of the International Health, Racquet, and Sportsclub Association confirmed that the highest number of gym membership sales is observed in January [26]. This phenomenon indicates that January might be a period of increased motivation for lifestyle changes. Importantly, previous reports state that the New Year’s resolution might a strong motivator, but the long-term effects of the decisions are poor [10, 27]. Therefore, an individual plans must include further follow-ups to maintain motivation of individuals. Index sales of most of the consumer goods decrease in January as compared to the previous month.

In February, students in Poland have two-weeks of winter holidays. This time may be associated with a decrease in sales due to travel abroad and family journeys. The forty days preceding Easter, known as Lent, begin at this time. Lent is traditionally related to personal resolutions and involves refraining from a pleasure or bad behavior [28, 29]. In Poland, almost 34 million, out of 38 million citizens, are Christian, mostly Roman Catholics [30]. Therefore, it may assumed that both the winter holidays and the tradition of Lent’s resolution may affect consumption. It was observed that in February, the sale of all types of products decreases as compared to previous months and this is consistent with national sale trends of non-durable consumer goods [31]. The decrease in sales was particularly notable for cigarette purchases. Furthermore, the interest in diet, dieticians, dietary supplements, weight loss, and gyms, remains high in comparison to the other months. Therefore, February in Poland, could be another month in which consumers have high motivation for lifestyle changes. In Croatia, there were massive public health campaigns encouraging smoke cessation during Lent with promising results [29, 32]. Such religiously-motivated interventions may be efficient, but further studies are needed to establish the same [33].

Base on the beginning date (Ash Wednesday), Lent lasts through March and even up to April, till Easter. In March and April, the increase in sales of sweets is most notable, and this may be associated with the tradition of gifting sweets to children for Easter [34]. As compared to February, purchases of other products also increase other than that of processed fishes and food fats, which is the lowest during April. It was observed that Polish Google users were still highly interested in lifestyle-related topics, but the relative search volumes decreased in comparison to January and February. The beginning of the spring may be the period when the hype of the New Year’s and Lent resolutions decreases. Therefore, it is crucial to communicate to individuals, who made a decision to change their lifestyle at the beginning of New Year or Lent, that March and April might be a period when motivation drops.

The highest interest in running is seen in May. Therefore, this period might be promising for pro-running health campaigns. Importantly, the campaign must involve weather forecasting to assist people to choose the optimal period for outdoor activities. At this time, the interest in other lifestyle-related topics continues to decrease from previous peaks, while the sales indexes of products consumed mostly in summer months increases. Furthermore, in May the activity of Polish Internet users is also slightly lower than that in colder months which is observed in monthly surveys of Polish Internet Researches (pl. Polskie Badania Internetu) [35].

The warmest period in Poland is from June to September. The summer holidays for schools begin in the last week of June till the end of August, while university students have an additional month of holidays, and the academic year begins in October. The consumption of ready-made meals, salty snacks, meats, beer, and cigarettes is the highest during these months. This a period of barbecue parties, holiday trips, and festivals, thus the sales of meat (e.g., sausages), snacks, alcohol, and cigarettes, increases [36]. Holiday activities may tempt individuals to follow an unhealthy diet pattern. Practitioners should encourage patients to maintain a healthy regime at this time. Skutecki et al. found that the triglycerides serum levels peak during summer months in Poland [37], and this may be because of the consumption of processed meat, snacks, and beer. Interestingly, the dairy sales index also peaks in warm months. The dairy consumption is most useful during cold months as there is limited insolation for Vitamin D. It is estimated that two-thirds of Poles suffer from Vitamin D deficiency [38]. This study speculates that cold dairy from a fridge may be tastier for consumers in summer months as during this period the consumption of dairy products increases [39]. However, it is crucial to promote dairy consumption during the cold season. Furthermore, the interest in healthy lifestyle-related topics is the lowest during the warm period. This drop may be associated with the lower time spent online during summer months as compare to the colder periods [35].

The second peak of interest in running is observed in summer. The weather is colder and people may be motivated to participate in outdoor activities after returning from holidays. This offers a potential period for the promotion of running.

The consumption of a number of goods decrease in autumn, but the purchase of sweets increases in comparison to summer. In Poland, sales of sweets are seen to peak before important holidays like Christmas and Easter [34], and during autumn [40]. Notably, most Polish people admit that they consume sweets compulsively and to improve their mood [41, 42]. Therefore, the increase in prevalence of depressive symptoms during this time may drive more people to consume sweets to improve their mood [43, 44]. Moreover, there is an increase in interest in diet, dietitian, dietary, and gyms in autumn, as compared to the summer. The weight gain during holidays could be motivation for a healthy lifestyle after people return to work or school [45]. Therefore, early autumn might be another optimal time for lifestyle interventions.

December is the month of Christmas and winter holidays. During this period, there is a peak in sales of processed fish and food fats. This may be related to the special Christmas time meals. The highest purchases of wine and strong alcohol could be associated with the New Year celebration [46]. Inversely, the interest of Google users in analyzed topics decreases during Christmas. These changes may be related to the lower activity of Web users, which has been suggested in past surveys [35, 47]. Christmas holidays can be a tough period for people who want to consume a healthy diet. Napierała et al. calculated that during Christmas, the average energy intake is approximately 2400 kcal, and the final weight gain after the holidays is estimated to be 420 g [48]. Moreover, an increase in total cholesterol levels has been observed after Christmas [37]. Therefore, it is important to educate the Polish community about traditional meals that should be preferred for health reasons. It may also be crucial to motivate people to increase their efforts to maintain good health after the Christmas holiday period ends and New Year begins.

Among food products, the most dynamic was the increase in sales of ready-made meals and salty snacks. The surge of consumption of processed foods might be related to an increase in unhealthy diet pattern among Polish adults observed in the last years [49]. The changes in sales of food products seem interesting, but they may be affected by the food supply, the introduction of new products, etc. Therefore, in our opinion, a new study should be performed to analyze secular trends in sales, and more chains should be compared.

The Polish Google users became more interested in searches related to dietitians and dietary supplements while less on physical activity and diet. Therefore, it may be related to unfavorable trends to search for an easier solution for losing weight or expansion of the dietary supplementary market and dietitians’ services. However, the problem requires further studies.

### Strengths and practical implications

As per our understanding, this is the first study that comprehensively investigates the seasonality of various lifestyle factors together. Previous research primarily focused on exercise and weight loss related searches in GT [19]. This study analyzes both, the sales indexes and the GT queries. It provides useful insights regarding consumption and the seasonal fluctuation in the interest in healthy habits. The findings may have practical implications. Furthermore, periods that might be optimal for lifestyle intervention for the entire population and at the individual level, have been identified. These findings may provide a background for future studies and for designing health promotion campaigns.

### Limitations

This study has several limitations. Firstly, the data does not present information about gender, age, and other characteristics of consumers and Google users. Secondly, commercial data has been collated from only one franchise, and the company did not disclose the number of purchases of consumer goods due to trade secret**.** Thirdly, the group of products is limited because not all facilities of the retail store chain include stands for fruits, vegetables, and organic (bio/eco) food. Fourthly, documentation about food supply and utilization has not been included. Limited food supply may affect sales indices. However, according to the store chain, “The food supply for the most popular products is practically not limited for franchisees, and we are rarely notified about problems with supplies. The more prevalent problem is a large amount of expired food which requires regular utilization” (personal communication, March 2021). Finally, the interest in different healthy behaviors among Google users does not mean that people tend to visit the gym, dietician, etc. more commonly. The search statistics might reflect general tendencies, but this is not the direct measurement of healthy behavior.

## Conclusion

In Poland, the purchase of food, drinks, and cigarettes, as well as the interest in information on different components of a healthy lifestyle displays seasonal variation. This observation may be useful for planning public health campaigns or for individual lifestyle interventions. New Year’s resolutions and Lent might be good periods to encourage healthy behavior. The motivation of people to maintaining a healthy lifestyle might decrease in summer and during Christmas, thus, individuals struggling with addictions or unhealthy habits should be taught how to resist temptation during these periods. Future real-world studies should verify how lifestyle interventions on an individual or populational level are affected by specific periods of the year.

## Supplementary Information

**Additional file 1.**

## Data Availability

The datasets used and/or analyzed during the current study are available on Mendeley Data (https://data.mendeley.com/datasets/3gsdt5t9pz/1): Kamiński, Mikołaj; Kręgielska-Narożna, Matylda; Bogdański, Paweł (2021), “Seasonal variation in lifestyle behavior in Poland: Google searches and market sales analysis”, Mendeley Data, V1, doi: 10.17632/3gsdt5t9pz.1 The dataset is publicly available. The data for Google Trends can be reproduced on: https://trends.google.pl/trends/ Google Trends is a publicly available website.

## Abbreviations

GT: Google Trends
RSV: Relative Search Volume
