# Supplementary material for: Seasonal variation in lifestyle behavior in Poland: Google searches and market sales analysis
Source: BMC Public Health. 2021 Aug 6;21:1516. doi: 10.1186/s12889-021-11543-9 (PMC8344181; doi:10.1186/s12889-021-11543-9)
Supplement: Supplementary file 1 — Additional file 1. [file 12889_2021_11543_MOESM1_ESM.doc]

Supplementary Table 1

Checklist for Documentation of Google Trends research.

| **Section/Topic** | **Checklist item** |
| --- | --- |
| **Search Variables** |  |
| Access Date | 29 February 2019 |
| Time Period | From January 2014 to August 2019 |
| Query Category | All |
| Region | Poland |
| **Search Input** | "Gym", "Running", "Weight loss", "Dietetic", "Dietary supplements", and "Diet" |
| **Rationale for Search Strategy** |  |
| For Search Input | The searched topics are related to healthy lifestyle |
| For Setting Chosen | The data was limited to Poland, because the study concern seasonal variation in this country. |
